# The potential mechanism of Fructus Ligustri Lucidi promoting osteogenetic differentiation of bone marrow mesenchymal stem cells based on network pharmacology, molecular docking and experimental identification

**DOI:** 10.1080/21655979.2022.2065753

**Published:** 2022-04-27

**Authors:** Yuanhang Kong, Xinnan Ma, Xin Zhang, Leilei Wu, Dechun Chen, Bo Su, Daqian Liu, Xintao Wang

**Affiliations:** Department of Orthopedic Surgery, The Second Affiliated Hospital of Harbin Medical University, Harbin, Heilongjiang, China

**Keywords:** Fructus Ligustri Lucidi, traditional Chinese medicine, bone marrow mesenchymal stem cells, osteogenic differentiation, PI3K/AKT signaling pathway, network pharmacology

## Abstract

Recent studies have shown that the differentiation of bone marrow mesenchymal stem cells (BMSCs) into osteogenic lineages can promotes bone formation and maintains bone homeostasis, which has become a promising therapeutic strategy for skeletal diseases such as osteoporosis. Fructus Ligustri Lucidi (FLL) has been widely used for the treatment of osteoporosis and other orthopedic diseases for thousands of years. However, whether FLL plays an anti-osteoporosis role in promoting the osteogenic differentiation of BMSCs, as well as its active components, targets, and specific molecular mechanisms, has not been fully elucidated. First, we obtained 13 active ingredients of FLL from the Traditional Chinese Medicine Systems Pharmacology (TCSMP) database, and four active ingredients without any target were excluded. Subsequently, 102 common drug-disease targets were subjected to protein-protein interaction (PPI) analysis, Gene Oncology (GO), and Kyoto Encyclopedia of Genes and Genomes (KEGG) enrichment analyses. The results of the three analyses were highly consistent, indicating that FLL promoted the osteogenic differentiation of BMSCs by activating the PI3K/AKT signaling pathway. Finally, we validated previous predictions using in vitro experiments, such as alkaline phosphatase (ALP) staining, alizarin red staining (ARS), and western blot analysis of osteogenic-related proteins. The organic combination of network pharmacological predictions with in vitro experimental validation comprehensively confirmed the reliability of FLL in promoting osteogenic differentiation of BMSCs. This study provides a strong theoretical support for the specific molecular mechanism and clinical application of FLL in the treatment of bone formation deficiency.

## GRAPHICAL ABSTRACT

**Figure:**
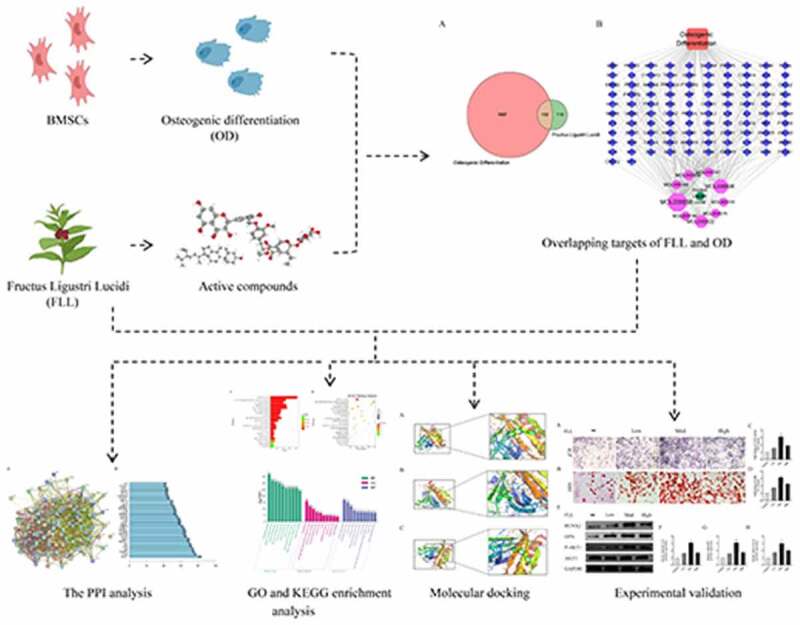

## Highlights

- Bone protective effects of FLL might result from achieved by promoting osteogenic differentiation of bone marrow mesenchymal stem cells (BMSCs).
- Beta-sitosterol, kaempferol, taxifolin, Lucidumoside D_qt, (20S)-24-ene-3β,20- diol-3-acetate, eriodictyol, syringaresinol diglucoside_qt, luteolin, quercetin might be the major active compounds of Fructus Ligustri Lucidi (FLL) promoting osteogenic differentiation of BMSCs.
- FLL might be target AKT1 and then activate the PI3K/AKT signaling pathway to promote the osteogenic differentiation of BMSCs.

## 1. Introduction

Bone marrow mesenchymal stem cells (BMSCs) play an important role in regenerative medicine, cell therapy, and tissue engineering which based on growth factors, scaffolds, and seed cells [1]. BMSCs have numerous advantages, such as rich sources, high survival rates, and rapid proliferation rates. Over the years, they have been widely used in the treatment of multiple diseases, particularly skeletal diseases. As we all know, BMSCs have the potential to differentiate into bone, cartilage, fat, and other directions [2]. During disease treatment, BMSCs can migrate to distant sites of tissue injury and exert a direct reparative effect through their differentiation [3]. In contrast, the decrease in proliferation and osteogenic differentiation of BMSCs may weaken bone homeostasis, thereby reducing the recovery ability of patients with skeletal diseases, such as osteoporosis and fractures [4,5]. Therefore, effective promotion of osteogenic differentiation of bone marrow mesenchymal stem cells may be a promising therapeutic strategy for related diseases characterized by an imbalance in bone homeostasis, such as osteoporosis [6].

Fructus Ligustri Lucidi (FLL), a traditional Chinese medicine, is obtained from the fruit of Ligustrum lucidum Ait, a member of the Oleaceae family, which is distributed in the Yangtze River Valley and south of China, including Zhejiang, Jiangsu, and Yunnan [7]. According to the literature, FLL has multiple biological activities, including anti-fatigue, anti-oxidation, anti-tumor, and anti-aging [8]. In addition, in Classic Materia Medica, FLL is described as a kind of herb with no obvious toxicity, indicating its high safety [9]. Therefore, FLL has been widely used in clinic for thousands of years, especially for the treatment of osteoporosis, fractures and other bone diseases [10]. One study showed that FLL could maintains the bone quality of aging osteoporosis mice by regulating intestinal microbial diversity, oxidative stress, and TMAO and SIRT6 levels [11]. Another study also confirmed that FLL may act as a natural antioxidant that inhibits the oxidative stress response and prevents the development of osteoporosis in OVX rats by regulating the Nox4/ROS/NF-κB signaling pathway [12].

Moreover, as the osteogenic differentiation of BMSCs plays a core role in maintaining bone homeostasis, the promotion of osteogenic differentiation of bone marrow mesenchymal stem cells has become a concern for an increasing number of researchers. The positive therapeutic effect of FLL on osteoporosis and other diseases with an imbalance of bone homeostasis has been confirmed. However, whether FLL exerts its therapeutic effect by promoting the osteogenic differentiation of BMSCs, as well as its active components, targets, and exact molecular mechanisms underlying the osteogenic differentiation of BMSCs, has not been fully elucidated.

As a result, our study is the first to identify potential bioactive compounds in FLL and elucidate its mechanisms in promoting the osteogenic differentiation of bone marrow mesenchymal stem cells by using the network pharmacology approach. The main goals of this study were to 1) screen potential FLL targets for promoting osteogenic differentiation in BMSCs; 2) use network pharmacology to analyze the underlying mechanisms of FLL promoting osteogenic differentiation in BMSCs; and 3) confirm the underlying pathway of FLL promoting osteogenic differentiation in BMSCs.The findings of this research might lead to a new therapeutic strategy for skeletal diseases. The graphical abstract depicts the present study’s technical plan.

## 2. Materials and methods

### 2.1. Determination of the active ingredients of FLL and prediction of its targets

The Traditional Chinese Medicine System Pharmacological analysis platform (TCMSP, http://lsp.nwu.edu.cn/tcmsp.php) is a unique database covering the chemical aspects of natural compounds, oral bioavailability, drug-likeness, intestinal epithelial permeability, and blood-brain barrier [13]. In this database, the active ingredients of FLL were obtained in strict accordance with the screening criteria: oral bioavailability (OB) ≥ 30% and drug-likeness (DL) ≥ 0.18 [14]. Simultaneously, the corresponding target proteins of each active ingredient screened according to the above criteria were acquired from the database. We then downloaded the Smiles structures files of the active ingredients from the PubChem database (https://pubchem.ncbi.nlm.nih.gov/) [15]. Moreover, the smile structures of the active ingredients were uploaded to the Swiss Target Prediction Network database (http://www.swisstargetprediction.ch/) to confirm the predicted target proteins of the active ingredients [16,17]. Finally, the names of the target proteins were unified using UniprotKB (https://www.uniprot.org/), duplicate items were deleted, and the data were combined and converted into gene symbols [18].

### 2.2. Prediction of osteogenic differentiation related targets

The target proteins related to osteogenic differentiation were respectively obtained from the GeneCards database (http://www.genecards.org/), and the Online Mendelian Inheritance in Man (OMIM) database (http://www.omim.org/) [19]. UniprotKB was also used to unify the names of target proteins, remove duplicate items, merge data, and convert gene symbols.

### 2.3. Establishment of FLL active ingredients and osteogenic differentiation interaction network

First, based on the above targets, we screened out the common targets of drugs and diseases and showed them using a Venn diagram. Second, we constructed a complex network of interactions among FLL, active ingredients, targets, and osteogenic differentiation. Finally, the network was analyzed, edited, and visualized using Cytoscape v3.8.2 (www.cytoscape.org/) [20].

### 2.4. Construction of PPI network and identification of hub genes

After converting the previously obtained common targets of drug and disease into gene symbols, these were inputted into String v11.0b (https://string-db.org/, updated on 17 October 2020) and searched using the “multiple proteins’ option to construct the corresponding PPI network [21]. Then, the results of the PPI network were saved in table text (TSV) format. Finally, R software (version 4.0.4) was used to analyze, calculate, and visualize the PPI results.

### 2.5. Common targets function enrichment analysis of FLL and osteogenic differentiation

KEGG pathway enrichment and GO enrichment analyses of the common targets were performed using the Metascape database (https://metascape.org/). Three types of GO enrichment analysis were performed: biological process (BP), molecular function (MF), and cellular components (CC). This method has been used to predict the specific molecular mechanism by which FLL promotes osteogenic differentiation [22]. The results were analyzed and collated, and graphs of enrichment GO terms, enrichment dot bubbles, and bars with color gradients were generated using http://www.bioinformatics.com.cn (an online platform for data analysis and visualization) [23].

### 2.6. Molecular docking

The interaction between the candidate active ingredients and key targets was further verified by molecular docking simulation, which provided a sufficient basis for FLL to promote osteogenic differentiation. Briefly, the SDF files of the three-dimensional chemical structures of candidate active ingredients were downloaded from the PubChem database, and the three-dimensional chemical structures were optimized using ChemBio3D Ultra 14.0 software. The structure of the target protein was obtained from the RCSB Protein Data Bank (PDB, http://www.pdb.org/) [24]. We then used AutoDockTools-1.5.6 and AutoDockVina-1.1.2 software to determine the active pocket of the target protein, and performed molecular docking between the candidate active ingredients and the target protein [25]. Finally, PyMoL-2.4.0 was used to analyze and visualize the binding mode and interactions of candidate active ingredients and key target proteins.

### 2.7. Isolation and culture of BMSCs

The BMSCs were extracted as previously described [26]. Briefly, Wistar rats (approximately 50 g) were sacrificed by CO_2_ inhalation and soaked in 75% alcohol for about 10 min. The hind limbs were transected along the groin, and soaked in 75% alcohol again for about 5 min. Soft tissues on the bone surface were carefully dissected under aseptic conditions and washed with sterile PBS. The epiphysis at both ends was excised, and the bone marrow cavity was exposed. The bone marrow cavity was repeatedly flushed with complete culture medium until its appearance changed from red to white. The liquid was collected in a 15 mL centrifuge tube and centrifuged centrifugation at 1000 r/min for 5 min. The supernatant was removed after centrifugation. Cells were resuspended in complete culture medium, then transferred into a T25 culture flask, and placed into incubator with volume fraction of 5% CO_2_, 37°C for culture. After two days, the solution was changed by half. Thereafter, the complete medium was changed every 2–3 days. Cell passage was performed when the cells reached 80%-90%. The third to fifth passages of the cells were used for subsequent experiments. This study was approved by the Ethics Committee of the Second Affiliated Hospital of Harbin Medical University (approval number KY2017-081).

### 2.8. Preparation of FLL aqueous extract and CCK-8 assay

FLL was acquired from a Chinese medicine store in Harbin, China. Raw FLL (250 g) was cooked twice in 4 L distilled water under reflux for 2 h. The filtered aqueous extracts were collected. The filtrate was lyophilized into a powder and concentrated under decreased pressure at 50°C. The yield of extraction was 20% (w/w). Chemical indicators oleanolic acid and ursolic acid had concentrations of 0.01% (w/w) and 0.015% (w/w), respectively. Prior to use, the extract powder was stored in a desiccator at room temperature . Third passage BMSCs were harvested and cultured in 96-well culture plates at 2 × 10^4^ cells/well. After 24 h of culture, the cells were divided into six groups: control group, FLL (25, 50, 100, 200, 400 µg/mL groups, and six wells in each group. Complete culture medium was added to the control group. All FLL groups were added to the complete culture medium with the corresponding concentration of FLL. After 0, 1, 2, and 3 days, one culture plate was removed for testing, and CCK-8 reagent (10 μL) was added to each well. The absorbance (A450 nm) value of BMSCs was detected using a microplate [27].

### 2.9. Alkaline phosphatase, Alizarin Red S staining and quantitative analysis

The third passage of BMSCs was seeded in 24-well culture plates at 5 × 10^4^ cells/well or 6-well culture plates at 1.5 × 10^5^ cells/well. The cells were treated with different concentrations of FLL at 80% confluence. The specific concentrations were as follows: FLL (Low, 25 µg/mL); FLL (Med, 50 µg/mL), and FLL (High, 100 µg/mL). Those not treated with FLL were used as control groups [28]. Next, 10 mM β-Glycerol-2-phosphate (Sigma-Aldrich, USA), 10 nM dexamethasone (Sigma-Aldrich, USA), and 50 mg/mL ascorbic acid (Sigma-Aldrich, USA) were added to each group. The culture medium was renewed every 2–3 days [29]. BMSCs were cultured for 7 days according to the above method. The culture medium was removed, and the cells were washed with PBS solution 2–3 times for 3–5 minutes each, fixed with 4% paraformaldehyde for 30 min, and washed with PBS solution 2–3 times for 3–5 minutes each. Cells were stained with BCIP/NBT Alkaline Phosphatase Kit (Beijing, Shanghai, China), digested, disrupted by sonication, and intracellular ALP activity was measured at 520 nm using an alkaline phosphatase activity assay kit. After 14 days of culture, BMSCs were washed and fixed as described above and then stained with Alizarin Red S (Cyagen, Guangzhou, China). An inverted microscope was used for image acquisition. The supernatant from each well was transferred to a 96-well plate after ARS was dissolved in cetylpyridinium chloride buffer (Sigma-Aldrich, USA). A microplate reader was used to read the OD value at 560 nm (Tecan Infinit M200, Switzerland) [30].

### 2.10. Western blot and quantitative analysis

According to a previously established method [31], RIPA (Beyotime, Shanghai, China) and PMSF (Beyotime, Shanghai, China) were prepared in working solution that was used to extract the total protein of BMSCs. Proteins were electrophoresed and resolved by SDS-PAGE gels, and PVDF membranes (Millipore, Billerica, MA, USA) were used to transfer the proteins on the gels. Primary antibodies against RUNX2 (Wanleibio, Shenyang, China), OPN (Wanleibio, Shenyang, China), AKT1 (Abcam, Cambridge, UK), P-AKT1 (Abcam, Cambridge, UK), and GAPDH (ABclonal, Wuhan, China) were diluted 1:1000 and incubated with PVDF membranes overnight at 4°C. After washing thrice with TBST, the PVDF membranes were incubated with the corresponding secondary antibodies (ABclonal, Wuhan, China) at a dilution ratio of 1:5000 for 1 h. Finally, proteins were visualized by chemiluminescence, and quantitatively analyzed using ImageJ software.

### 2.11. Reverse-transcriptase-polymerase chain reaction (RT-PCR)

The mRNA expression levels of RUNX2, OPN, and AKT1 were determined using quantitative real-time PCR [32]. After osteogenic stimulation for 7 days, total RNA was extracted from the cells with TRIzol (Thermo, USA) and reverse transcribed into cDNA using a ReverTra Ace qPCR RT Kit (ABclonal, Wuhan, China), following the manufacturer’s instructions. Quantitative real-time PCR was carried out using SYBR Green PCR Master Mix (ABclonal, Wuhan, China) under the following thermocycling conditions: 95°C for 30s and 40 cycles at 95°C for 5 s and 55°C for 30s. Specific primer sequences are listed in Table 1. GAPDH was used as the internal control for PCR. The relative mRNA expression was calculated using the 2^−ΔΔCt^ method.

**Table 1.** PCR primer sequences

Gene	Primer Sequences	
RUNX2	Forward	$5{\;}^{\prime }$-TGGCCTTCCTCTCTCAGTAA-3’
	Reverse	$5{\;}^{\prime }$-GTAAGTGAAGGTGGCTGGATAG-3’
OPN	Forward	$5{\;}^{\prime }$-TGAGTTTGGCAGCTCAGAGGAGAA-3’
	Reverse	$5{\;}^{\prime }$ ‘ATCATCGTCCATGTGGTCATGGCT-3’
AKT1	Forward	$5{\;}^{\prime }$- AGCGACGTGGCTATTGTGAAG −3’
	Reverse	$5{\;}^{\prime }$- GCCATCATTCTTGAGGAGGAAGT-3’
GAPDH	Forward	$5{\;}^{\prime }$- GGAGCGAGATCCCTCCAAAAT −3’
	Reverse	$5{\;}^{\prime }$- GGCTGTTGTCATACTTCTCATGG −3’

### 2.12. Statistical analysis

GraphPad Prism (version 8.1) software was used for statistical analysis. All experiments were independently repeated at least three times. The results are expressed as mean ± standard deviation (SD). One Way ANOVA was used for comparison between groups. P < 0.05 was considered statistically significant.

## 3. Results

Based on pharmacological and experimental validation, the goal of this study was to investigate the pharmacological mechanisms of FLL in boosting the osteogenic differentiation of BMSCs. There are three aspects to this approach. First, we examined FLL’s active components and their probable targets, and the OMIM and Genecards databases were used to identify osteogenic differentiation targets. Second, the target genes of FLL that promote BMSCs osteogenic development were assessed, followed by GO and KEGG enrichment analyses. Third, for molecular docking, AutoDock Tools 1.1.2 was utilized. Finally, experimental evidence shows that FLL activated the PI3K/AKT signaling pathway, promoting the osteogenic differentiation of BMSCs. Finally, we used in vitro tests, such as alkaline phosphatase staining, alizarin red staining, western blot analysis of osteogenic-related proteins, and PCR to confirm earlier predictions.

### 3.1. Active ingredients and targets of FLL

Strictly following the screening criteria of oral bioavailability (OB) ≥ 30% and drug-likeness (DL) ≥ 0.18, we acquired 13 active ingredients of FLL (beta-sitosterol, kaempferol, taxifolin, lucidumoside D, lucidumoside D-qt, (20S)-24-ene-3β,20-diol-3-acetate, eriodictyol, syringaresinol diglucoside_qt,lucidusculine,olitoriside, olitoriside-qt, luteolin, and quercetin) from the TCMSP database (Table 2). Because no target proteins related to lucidumoside D, lucidusculine, olitoriside, and olitoriside-qt were found, these four active ingredients were excluded from this study. The target proteins of FLL were accurately predicted using the TCMSP and Swiss Target Prediction Network databases. A total of 215 target proteins were obtained after deleting repetitive items.

**Table 2.** 13 main active ingredients of FLL

Molecule ID	Molecule name	Molecular weight	OB (%)	DL
MOL000358	beta-sitosterol	414.79	36.91	0.75
MOL000422	kaempferol	286.25	41.88	0.24
MOL004576	taxifolin	304.27	57.84	0.27
MOL005146	Lucidumoside D	568.63	48.87	0.71
MOL005147	Lucidumoside D_qt	406.47	54.41	0.47
MOL005169	(20S)-24-ene-3β,20-diol-3-acetate	486.86	40.23	0.82
MOL005190	eriodictyol	288.27	71.79	0.24
MOL005195	syringaresinol diglucoside_qt	450.48	83.12	0.80
MOL005209	Lucidusculine	401.6	30.11	0.75
MOL005211	Olitoriside	696.87	65.45	0.23
MOL005212	Olitoriside_qt	404.55	103.23	0.78
MOL000006	luteolin	286.25	36.16	0.25
MOL000098	quercetin	302.25	46.43	0.28

### 3.2. Osteogenic differentiation related targets

We combined the GeneCards and OMIM databases to screen osteogenic differentiation-related targets as comprehensively as possible, analyzed the data, removed duplicate target proteins, and finally obtained 1739 target proteins related to osteogenic differentiation.

### 3.3. Prediction of potential targets of FLL promoting osteogenic differentiation and construction of FLL-targets-osteogenic differentiation interaction network

**Figure 1.**
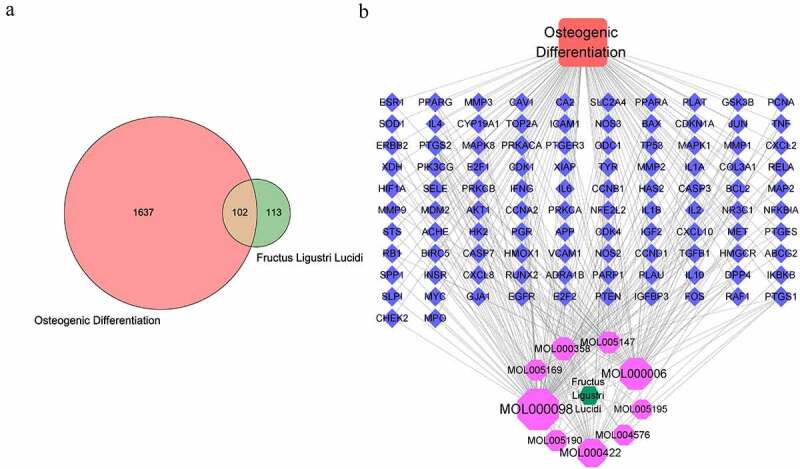
The targets of FLL and osteogenic differentiation and interaction network. (a) 102 common targets between FLL and osteogenic differentiation. (b) FLL-targets-osteogenic differentiation network analysis.

By analyzing the related targets of FLL and osteogenic differentiation, it is reasonable to believe that the intersection items are the potential targets of FLL promoting osteogenic differentiation, which are displayed through a Venn diagram. There were 102 common targets that can be considered key targets of FLL in promoting osteogenic differentiation (Figure 1a). We present an interaction network of FLL-target-osteogenic differentiation (Figure 1b). FLL and osteogenic differentiation are indicated by green and red nodes, respectively. Nine purple nodes represent the active ingredients of FLL, and 102 blue nodes represent the common targets of FLL and osteogenic differentiation. The edges represent the mutual interactions between various nodes. This network further indicated that FLL may play a role in the osteogenic differentiation of BMSCs through the positive or negative regulation of these targets.

### 3.4. Determination of PPI network and hub genes

**Figure 2.**
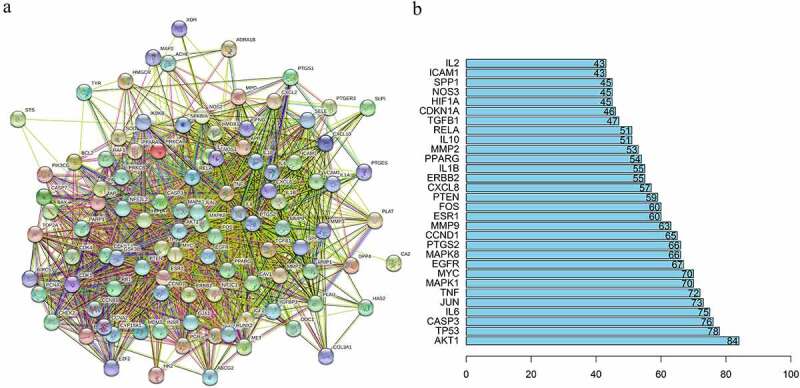
PPI network and hub genes. (a) The PPI network of common targets of FLL and osteogenic differentiation. (b) The bar plot of top 30 hub genes.

PPI analysis of 102 common targets of FLL and osteogenic differentiation using the STRING database resulted in a complex network consisting of 102 nodes and 1757 edges (Figure 2a). Nodes represent proteins, and edges represent interactions between nodes. The greater the number of edges connected to a node, the more important is the biological function of that node in the network, and is considered a hub gene. The number of edges connected to each node was analyzed and calculated using the R software, and the top 30 hub genes were selected to create a bar plot (Figure 2b). We found that RAC-alpha-serine/threonine-protein kinase (AKT1) was the first of these 30 hub genes, indicating that FLL may regulate the osteogenic differentiation of BMSCs through this target.

### 3.5. Biological functions of common targets of FLL and osteogenic differentiation

**Figure 3.**
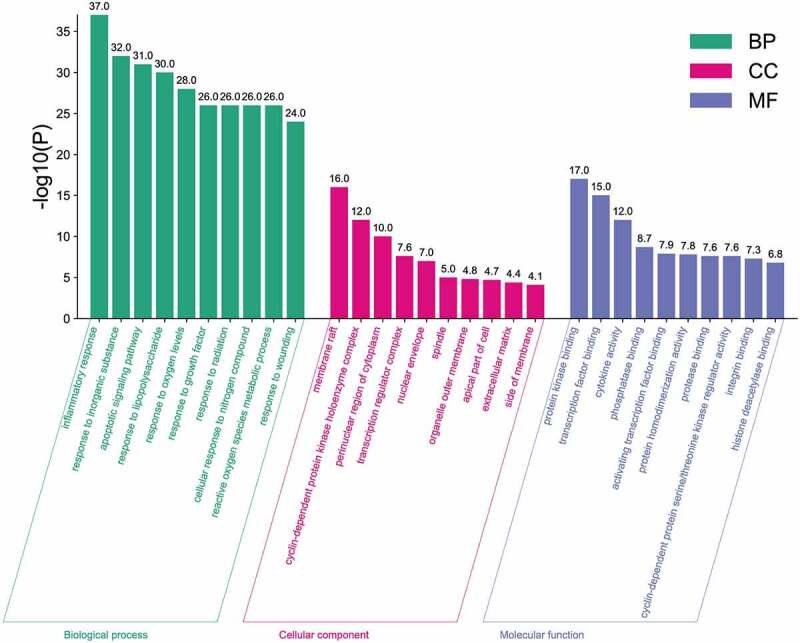
GO analysis. The X-axis represents three GO Terms: biological processes (BP), cellular components (CC), and molecular functions (MF). The y-axis represents -log10(p) of GO terms. The -log (P) value of GO Terms was represented numerically above the bar plot.

**Figure 4.**
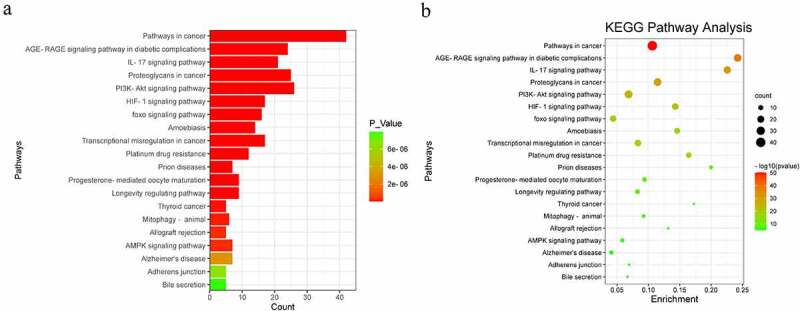
KEGG pathway analysis. (a) Bar plot of KEGG pathway analysis. The X-axis represents the number of genes enriched into the pathway, from green to red representing the change of P value. (b) Dot plot of KEGG pathway analysis. The number of genes enriched into each pathway is represented by the circle size, from green to red representing the change of P value.

To further illustrate the biological functions of common targets, we performed GO and KEGG enrichment analyses of these targets using the Metascape database. GO enrichment analysis histograms were plotted with GO terms as the X-axis and −log10(P) as the Y-axis, target-related biological processes (BP), cellular components (CC), and molecular functions (MF) represented by green, purple, and blue columns, respectively [33]. We found that inflammatory response (37.0), membrane raft (16.0), and protein kinase binding (17.0) were the most representative GO terms in BP, CC, and MF, respectively (Figure 3). In addition, 102 common targets of FLL and osteogenic differentiation were enriched in 166 KEGG pathways, and important targets were mainly enriched in the PI3K/AKT signaling pathway (Figure 4(a–b)), indicating that FLL promotes osteogenic differentiation of BMSCs by activating the PI3K/AKT signaling pathway.

### 3.6. Molecular docking of AKT1 to the main active ingredients of FLL

**Figure 5.**
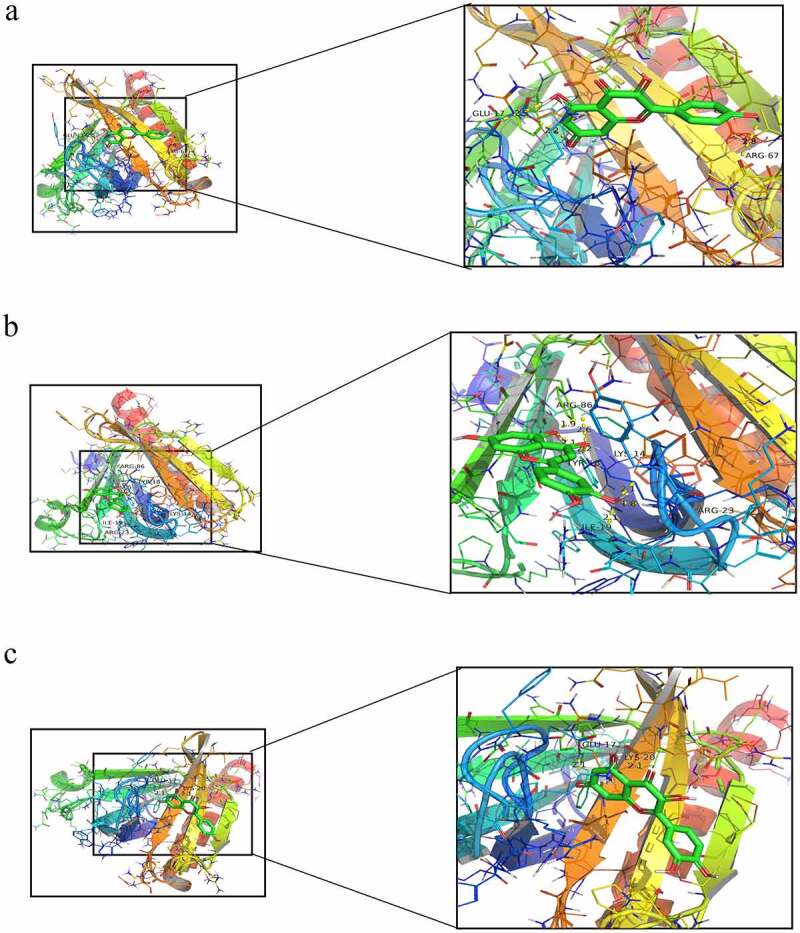
The molecular docking study of main active ingredients and core targets. (a) AKT1 with kaempferol. (b) AKT1 with luteolin. (c) AKT1 with quercetin. Ball-and-stick model represented molecules, dotted line represented hydrogen bonds, and the distance is in angstroms.

Based on the prediction that AKT1 is the core target of FLL in promoting osteogenic differentiation, we selected three active ingredients of FLL (kaempferol, luteolin, and quercetin), which contain AKT1 as the target, for molecular docking analysis, to investigate the potential way in which FLL exerts its therapeutic effect through AKT1. Our results suggest that the interaction between FLL and AKT1 may be determined by hydrogen bonds formed between kaempferol and residues GLU-17 and ARG-67 in AKT1 (Figure 5a); luteolin and residues ARG-86, TYR-18, LYS-14, ARG-23, and ILE-19 in AKT1 (Figure 5b), as well as quercetin and residues GLU-17 and LYS-20 in AKT1 (Figure 5c).

### 3.7. Effects of FLL on BMSCs proliferation

Compared with the control group, FLL at concentrations of 25, 50, and 100 µg/mL stimulated the proliferation of BMSCs. Among them, the 50 µg/mL and 100 μg/mL groups had the most significant effects of promoting proliferation, but there was no statistical difference between the two groups. However, FLL had a significant inhibitory effect on BMSCs at concentrations of ≥ 200 µg/mL (Figure S1). Therefore, we chose the FLL group, which had no inhibitory effect on BMSCs, for subsequent experiments.

### 3.8. FLL promotes ALP activity, calcium deposit formation, and expression of osteogenic-related proteins by activating PI3K/AKT signaling pathway

**Figure 6.**
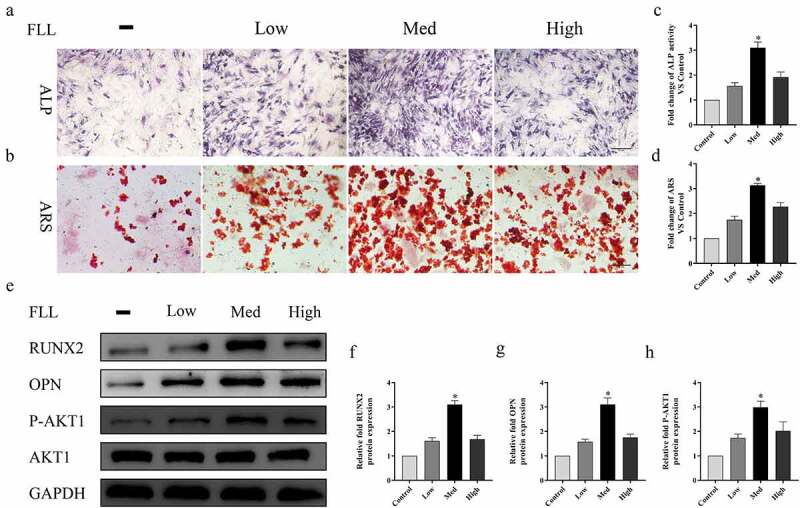
FLL promotes osteogenic differentiation of BMSCs by activating the PI3K/AKT signaling pathway. (a) ALP straining at 7 days. (b) ARS straining at 14 days. (c) Western blot analyses of P-AKT1, RUNX2 and OPN at 7 days. (d–f) Relative quantitative analysis. *P < 0.05 compared with control group. Scale bar = 10 μm; magnification, × 40.

The results of alkaline phosphatase staining on the 7th day show that the expression of alkaline phosphatase in each group was increased to different extents after FLL treatment compared with the control group. In particular, the stained areas and depth of color in the FLL (Med, 50 µg/mL) group were significantly higher than those in the other groups (Figure 6a). Calcium deposit formation was detected by ARS staining on day 14. The results show that more calcium deposits formed in the FLL (Med, 50 µg/mL) group, which is consistent with the results of ALP staining (Figure 6b). Quantitative analysis of ALP and ARS shows the same trend as the above staining (Figure 6(c–d)). Western blotting and quantitative analysis on day 7 show that FLL treatment increased the expression of RUNX2, OPN, and P-AKT1 to varying degrees, and the protein expression in the FLL (Med, 50 µg/mL) group was the highest, consistent with the ALP and ARS results (Figure 6(e–h)).

### 3.9. Inhibition of the PI3K/AKT signaling pathway blocked the FLL-mediated osteogenic differentiation of BMSCs

**Figure 7.**
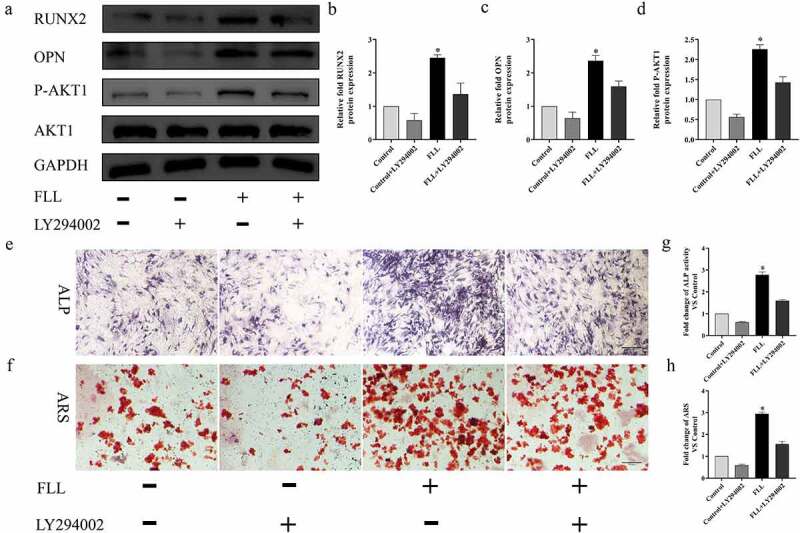
The enhancement of osteogenic differentiation of BMSCs induced by FLL was partially blocked by PI3K/AKT signal pathway inhibitors. (a) The protein expression levels of P-AKT1, RUNX2 and OPN treatment of LY294002 (20 µM) for 7 days. (b–d) Relative quantitative analysis of protein expression levels. (e) Results of ALP staining. (f) Results of ALP staining. *P < 0.05 vs control group. Scale bar = 10 μm; magnification, × 40.

We tested the inhibitory effect of the PI3K/AKT signaling pathway on osteogenic differentiation using a PI3K/AKT inhibitor (LY294002) to verify whether FLL promoted osteogenic differentiation of BMSCs through this signaling pathway [34]. We found that the increase in protein expression levels of RUNX2, OPN, and P-AKT1 induced by FLL (Med, 50 µg/mL) was partially inhibited by the addition of LY2940027 for 7 days (Figure 7(a–d)). In addition, ALP and ARS staining and quantitative analysis results also show that LY294002 partially blocked the FLL-mediated osteogenic differentiation of BMSCs by inhibiting the PI3K/AKT signaling pathway (Figure 7(e–h)).

### 3.10. Effects of FLL on the mRNA levels of RUNX2, OPN, and AKT1

The mRNA levels of AKT1 and the osteogenic genes RUNX2 and OPN were higher in the FLL group than in the control group (P < 0.05). The mRNA levels of AKT1 and the osteogenic genes RUNX2 and OPN in the FLL group were also higher than those in the FLL+ LY294002 group (P < 0.05) (Figure S2).

## 4. Discussion

BMSCs are driven by different signaling molecules and can differentiate into bone, cartilage, and fat which fully reflects the multidirectional differentiation of these cells. The impaired ability of BMSCs to differentiate into different lineages can lead to a variety of diseases including aging and osteoporosis [35]. The weakening of the differentiation ability of BMSCs into the osteogenic lineage can lead to insufficient bone formation and weaken bone homeostasis, resulting in skeletal diseases such as osteoporosis, which seriously threatens the physical and mental health of people worldwide [36]. Therefore, promoting osteogenic differentiation of BMSCs is a novel and effective therapeutic strategy for diseases such as osteoporosis [37]. TCM has few toxic side effects and a wide range of sources, and is a good natural product for the treatment of bone diseases such as osteoporosis and fractures, and have been used for thousands of years. In recent years, increasing attention has been paid to the anti-osteoporotic effects of TCM based on the regulation of the osteogenic differentiation of BMSCs [38]. The results of one study confirmed the molecular mechanism by which genistein promotes osteogenic differentiation through the BMP2/Smad5/Runx2 signaling pathway [39]. In addition, medicarpin targets estrogen receptor beta to stimulate osteoblast differentiation, and icariin promotes the osteogenic differentiation of BMSCs by enhancing the expression of BMAL1 through BMP signaling [40, 41].

The effectiveness of FLL in the treatment of skeletal diseases such as osteoporosis has been confirmed by numerous studies [10–12]. However, few studies have reported how FLL affects the osteogenic differentiation of BMSCs. In particular, the specific molecular mechanism by which FLL promotes osteogenic differentiation of BMSCs has not been fully clarified. Therefore, we combined network pharmacological analysis with in vitro experiments, such as alkaline phosphatase staining, alizarin red staining, western blotting, to comprehensively elucidate the mechanism of by which FLL promotes osteogenic differentiation of BMSCs. To our knowledge, this is the first study to investigate the mechanism by which FLL promotes osteogenic differentiation of BMSCs using a combination of bioinformatics and in vitro experiments. Using this approach, we found that FLL may exert positive effects on the osteogenic differentiation of BMSCs through its 9 major active ingredients acting on 102 common targets of drugs and diseases. Subsequently, we further investigated the main biological functions of these 102 targets by PPI and enrichment analyses. The top 30 targets (AKT1, TP53, CASP3, IL6, JUN, TNF, MAPK1, MYC, EGFR, MAPK8, PTGS2, CCND1, MMP9, ESR1, FOS, PTEN, CXCL8, ERBB2, IL1B, PPARG, MMP2, IL10, RELA, TGFB1, CDKN1A, HIF1A, NOS3, SPP1, ICAM1, and IL2) were identified. AKT1 was located at the core of this network, with a score of 84, and was considered most likely to be the main target of FLL exerting biological functions. The results of the GO enrichment analysis show that the representative GO term in MF was protein kinase binding (17.0). Coincidentally, AKT1, the most central target in our PPI network, is an important member of the protein kinase family, reinforcing our conclusion that AKT1 is a core target of FLL in the promotion of the osteogenic differentiation of BMSCs. The results of the KEGG pathway enrichment analysis were equally surprising, with 166 pathways being enriched, and the PI3K/AKT signaling pathway (hsa04151), among the signaling pathways associated with osteogenic differentiation, showed the best enrichment effect. In summary, the highly consistent results of PPI network, GO, and KEGG pathway enrichment analyses fully illustrate the reliability of FLL in promoting osteogenic differentiation of BMSCs by acting on AKT1 to activate the PI3K/AKT signaling pathway. Moreover, in our study, the molecular docking simulation results with multiple active ingredients of FLL (kaempferol, luteolin, and quercetin) and AKT1 demonstrate a reliable relationship between FLL and the core target AKT1 from another perspective.

It has been reported that tyrosine and serine/threonine kinases are indispensable in the process of cell differentiation resulting from extracellular factors, such as drugs and growth factors [42,43]. As a serine/threonine kinase, the pleckstrin homology domain of AKT binds to PtdIns (3,4,5) P3 to rapidly increase its expression in cells stimulated by drugs and other external factors, leading to AKT activation through phosphorylation, which promotes cell differentiation after activation of the PI3K/AKT signaling pathway [44,45]. A previous study described a severe delay in bone development by establishing a model of AKT1/AKT2 knockout mice, which fully shows the importance of AKT in bone formation [46]. Our study confirmed that FLL (Med, 50 µg/mL) significantly increased the expression of P-AKT1 and activated the PI3K/AKT signaling pathway to promote osteogenic differentiation of BMSCs. The detection of osteogenic markers such as ALP, ARS, RUNX2 and OPN also successfully demonstrated the effectiveness of FLL on osteogenic differentiation of BMSCs. These results are consistent with those of other studies [28]. The classical inhibitor LY294002 decreased FLL-induced expression of P-AKT1. ALP and ARS staining and the expression of RUNX2 and OPN proteins were also inhibited, further indicating that the PI3K/AKT signaling pathway is involved in the FLL-mediated osteogenic differentiation of BMSCs [47–49].

In summary, we comprehensively verified the molecular mechanism by which FLL promotes the osteogenic differentiation of BMSCs through activation of the PI3K/AKT signaling pathway through a combination of network pharmacological prediction as well as in vitro experiments. However, more in-depth research is required. Nevertheless, our study provides new insights into the treatment of bone formation deficiency diseases by FLL, based on the new perspective of promoting the osteogenic differentiation of BMSCs.

## 5. Conclusions

In this study, we combined network pharmacological prediction and in vitro experimental validation to confirm that FLL acts on AKT1 through a variety of effective active ingredients and promote BMSCs osteogenic differentiation by activating the PI3K/AKT signaling pathway. This provides a valuable theoretical basis for the specific molecular mechanism and clinical application of FLL in the treatment of bone formation deficiency diseases, aid in FLL drug screening, and serve as a helpful reference for the investigation of other TCM therapeutic mechanisms.

## Supplementary Material

Supplemental Material

## Data Availability

The data related to this study can be obtained from the corresponding author upon reasonable request.

## References

[1] Fröhlich M, Grayson WL, Wan LQ, et al. Tissue engineered bone grafts: biological requirements, tissue culture and clinical relevance. Curr Stem Cell Res Ther. 2008;3(4):254–264. 19075755 10.2174/157488808786733962 PMC2773298

[2] Matsushita K. Mesenchymal stem cells and metabolic syndrome: current understanding and potential clinical implications. Stem Cells Int. 2016;2016:2892840. 27313625 10.1155/2016/2892840 PMC4903149

[3] Polymeri A, Giannobile WV, Kaigler D. Bone marrow stromal stem cells in tissue engineering and regenerative medicine. Horm Metab Res. 2016;48(11):700–713. 27871114 10.1055/s-0042-118458 PMC9284410

[4] Cui H, Han G, Sun B, et al. Activating PIK3CA mutation promotes osteogenesis of bone marrow mesenchymal stem cells in macrodactyly. Cell Death Dis. 2020;11(7):505. 32632138 10.1038/s41419-020-2723-6 PMC7338441

[5] Egermann M, Schneider E, Evans CH, et al. The potential of gene therapy for fracture healing in osteoporosis. Osteoporos Int. 2005;16(Suppl 2):S120–S128. 15654580 10.1007/s00198-004-1817-9

[6] Li Y, Feng C, Gao M, et al. MicroRNA-92b-5p modulates melatonin-mediated osteogenic differentiation of bone marrow mesenchymal stem cells by targeting ICAM-1. J Cell Mol Med. 2019;23(9):6140–6153. 31304676 10.1111/jcmm.14490 PMC6714169

[7] He F, Chen L, Liu Q, et al. Preparative separation of phenylethanoid and secoiridoid glycosides from ligustri lucidi fructus by high-speed counter-current chromatography coupled with ultrahigh pressure extraction. Molecules. 2018;23(12):3353. 10.3390/molecules23123353 PMC6321428 30567348

[8] Li ZY, Li Q, Lü J, et al. LC-MS determination and pharmacokinetic study of salidroside in rat plasma after oral administration of suspensions of traditional Chinese medicine Erzhi Wan and Fructus Ligustri lucidi. J Pharm Anal. 2011;1(1):8–12. 29403675 10.1016/S2095-1779(11)70002-8 PMC5760783

[9] Chen B, Wang L, Li L, et al. Fructus Ligustri Lucidi in osteoporosis: a review of its pharmacology. Phytochemistry, Pharmacokinetics Safety Molecules. 2017;22(9):1469. 10.3390/molecules22091469 PMC6151717 28872612

[10] Wang L, Ma R, Guo Y, et al. Antioxidant effect of Fructus Ligustri Lucidi Aqueous extract in ovariectomized rats is mediated through Nox4-ROS-NF-κB pathway. Front Pharmacol. 2017;8:266. 28588482 10.3389/fphar.2017.00266 PMC5438993

[11] Li L, Chen B, Zhu R, et al. Fructus Ligustri Lucidi preserves bone quality through the regulation of gut microbiota diversity, oxidative stress, TMAO and Sirt6 levels in aging mice. Aging (Albany NY). 2019;11(21):9348–9368. 31715585 10.18632/aging.102376 PMC6874471

[12] Ko CH, Siu WS, Lau CP, et al. Osteoprotective effects of Fructus Ligustri Lucidi aqueous extract in aged ovariectomized rats. Chin Med. 2010;5:39. 21114821 10.1186/1749-8546-5-39 PMC3003250

[13] Yang S, Zhang J, Yan Y, et al. Network pharmacology-based strategy to investigate the pharmacologic mechanisms of atractylodes macrocephala Koidz. for the treatment of chronic gastritis. Front Pharmacol. 2020;10:1629. 32063848 10.3389/fphar.2019.01629 PMC7000373

[14] Hu S, Chen S, Li Z, et al. Research on the potential mechanism of Chuanxiong Rhizoma on treating diabetic nephropathy based on network pharmacology. Int J Med Sci. 2020;17(15):2240–2247. 32922187 10.7150/ijms.47555 PMC7484651

[15] Qi P, Li J, Gao S, et al. Network pharmacology-based and experimental identification of the effects of quercetin on Alzheimer’s disease. Front Aging Neurosci. 2020;12:589588. 33192484 10.3389/fnagi.2020.589588 PMC7645061

[16] Daina A, Michielin O, Zoete V. SwissTargetPrediction: updated data and new features for efficient prediction of protein targets of small molecules. Nucleic Acids Res. 2019;47(W1):W357–W364. 31106366 10.1093/nar/gkz382 PMC6602486

[17] Gfeller D, Michielin O, Zoete V. Shaping the interaction landscape of bioactive molecules. Bioinformatics. 2013;29(23):3073–3079. 24048355 10.1093/bioinformatics/btt540

[18] Fu SQ, Wang ZY, Jiang ZM, et al. Integration of zebrafish model and network pharmacology to explore possible action mechanisms of morinda officinalis for treating osteoporosis. Chem Biodivers. 2020;17(5):e2000056. 32190963 10.1002/cbdv.202000056

[19] Liu WJ, Jiang ZM, Chen Y, et al. Network pharmacology approach to elucidate possible action mechanisms of Sinomenii Caulis for treating osteoporosis. J Ethnopharmacol. 2020;257:112871. 32325182 10.1016/j.jep.2020.112871

[20] Shannon P, Markiel A, Ozier O, et al. Cytoscape: a software environment for integrated models of biomolecular interaction networks. Genome Res. 2003;13(11):2498–2504. 14597658 10.1101/gr.1239303 PMC403769

[21] Szklarczyk D, Gable AL, Lyon D, et al. STRING v11: protein-protein association networks with increased coverage, supporting functional discovery in genome-wide experimental datasets. Nucleic Acids Res. 2019;47(D1):D607–D613. 30476243 10.1093/nar/gky1131 PMC6323986

[22] Zhou Y, Zhou B, Pache L, et al. Metascape provides a biologist-oriented resource for the analysis of systems-level datasets. Nat Commun. 2019;10(1):1523. 30944313 10.1038/s41467-019-09234-6 PMC6447622

[23] Niu L, Dang C, Li L, et al. Next-generation sequencing-based identification of EGFR and NOTCH2 complementary mutations in non-small cell lung cancer. Oncol Lett. 2021;22(2):594. 34149905 10.3892/ol.2021.12855 PMC8200943

[24] Burley SK, Bhikadiya C, Bi C, et al. RCSB protein data bank: powerful new tools for exploring 3D structures of biological macromolecules for basic and applied research and education in fundamental biology, biomedicine, biotechnology, bioengineering and energy sciences. Nucleic Acids Res. 2021;49(D1):D437–D451. 33211854 10.1093/nar/gkaa1038 PMC7779003

[25] Trott O, Olson AJ. AutoDock Vina: improving the speed and accuracy of docking with a new scoring function, efficient optimization, and multithreading. J Comput Chem. 2010;31(2):455–461. 19499576 10.1002/jcc.21334 PMC3041641

[26] Li Q, Jin Y, Ye X, et al. Bone marrow mesenchymal stem cell-derived exosomal MicroRNA-133a restrains myocardial fibrosis and epithelial-mesenchymal transition in viral myocarditis rats through suppressing MAML1. Nanoscale Res Lett. 2021;16(1):111. 34215939 10.1186/s11671-021-03559-2 PMC8253878

[27] Hann SY, Cui H, Esworthy T, et al. Dual 3D printing for vascularized bone tissue regeneration. Acta Biomater. 2021;123:263–274. 33454383 10.1016/j.actbio.2021.01.012

[28] Li G, Zhang XA, Zhang JF, et al. Ethanol extract of Fructus Ligustri Lucidi promotes osteogenesis of mesenchymal stem cells. Phytother Res. 2010;24(4):571–576. 19813230 10.1002/ptr.2987

[29] Smith BT, Bittner SM, Watson E, et al. Multimaterial dual gradient three-dimensional printing for osteogenic differentiation and spatial segregation. Tissue Eng Part A. 2020;26(5–6):239–252. 31696784 10.1089/ten.tea.2019.0204 PMC7133451

[30] Bai J, Xu J, Hang K, et al. Glycyrrhizic acid promotes osteogenic differentiation of human bone marrow stromal cells by activating the Wnt/β-Catenin signaling pathway. Front Pharmacol. 2021;12:607635. 33935702 10.3389/fphar.2021.607635 PMC8085383

[31] Zhong W, Li X, Pathak JL, et al. Dicalcium silicate microparticles modulate the differential expression of circRNAs and mRNAs in BMSCs and promote osteogenesis via circ_1983-miR-6931-Gas7 interaction. Biomater Sci. 2020;8(13):3664–3677. 32463418 10.1039/d0bm00459f

[32] Livak KJ, Schmittgen TD. Analysis of relative gene expression data using real-time quantitative PCR and the 2(-Delta Delta C(T)) method. Methods. 2001;25(4):402–408. 11846609 10.1006/meth.2001.1262

[33] Xu L, Yang P, Feng Y, et al. Spatiotemporal transcriptome analysis provides insights into bicolor tepal development in Lilium “Tiny Padhye”. Front Plant Sci. 2017;8:398. 28392796 10.3389/fpls.2017.00398 PMC5364178

[34] Mukherjee A, Rotwein P. Akt promotes BMP2-mediated osteoblast differentiation and bone development. J Cell Sci. 2009;122(Pt 5):716–726. 19208758 10.1242/jcs.042770 PMC2720922

[35] Zou D, Han W, You S, et al. In vitro study of enhanced osteogenesis induced by HIF-1α-transduced bone marrow stem cells. Cell Prolif. 2011;44(3):234–243. 21535264 10.1111/j.1365-2184.2011.00747.x PMC6496451

[36] Pino AM, Rosen CJ, Rodríguez JP. In osteoporosis, differentiation of mesenchymal stem cells (MSCs) improves bone marrow adipogenesis. Biol Res. 2012;45(3):279–287. 23283437 10.4067/S0716-97602012000300009 PMC8262098

[37] Chen Q, Shou P, Zheng C, et al. Fate decision of mesenchymal stem cells: adipocytes or osteoblasts? Cell Death Differ. 2016;23(7):1128–1139. 26868907 10.1038/cdd.2015.168 PMC4946886

[38] Cheng YH, Dong JC, Bian Q. Small molecules for mesenchymal stem cell fate determination. World J Stem Cells. 2019;11(12):1084–1103. 31875870 10.4252/wjsc.v11.i12.1084 PMC6904864

[39] Dai J, Li Y, Zhou H, et al. Genistein promotion of osteogenic differentiation through BMP2/SMAD5/RUNX2 signaling. Int J Biol Sci. 2013;9(10):1089–1098. 24339730 10.7150/ijbs.7367 PMC3858582

[40] Bhargavan B, Singh D, Gautam AK, et al. Medicarpin, a legume phytoalexin, stimulates osteoblast differentiation and promotes peak bone mass achievement in rats: evidence for estrogen receptor β-mediated osteogenic action of medicarpin. J Nutr Biochem. 2012;23(1):27–38. 21333515 10.1016/j.jnutbio.2010.11.002

[41] Huang Z, Wei H, Wang X, et al. Icariin promotes osteogenic differentiation of BMSCs by upregulating BMAL1 expression via BMP signaling. Mol Med Rep. 2020;21(3):1590–1596. 32016461 10.3892/mmr.2020.10954 PMC7002972

[42] Ghosh-Choudhury N, Abboud SL, Nishimura R, et al. Requirement of BMP-2-induced phosphatidylinositol 3-kinase and Akt serine/threonine kinase in osteoblast differentiation and Smad-dependent BMP-2 gene transcription. J Biol Chem. 2002;277(36):33361–33368. 12084724 10.1074/jbc.M205053200

[43] Qiao J, Paul P, Lee S, et al. PI3K/AKT and ERK regulate retinoic acid-induced neuroblastoma cellular differentiation. Biochem Biophys Res Commun. 2012;424(3):421–426. 22766505 10.1016/j.bbrc.2012.06.125 PMC3668681

[44] Hidaka K, Kanematsu T, Takeuchi H, et al. Involvement of the phosphoinositide 3-kinase/protein kinase B signaling pathway in insulin/IGF-I-induced chondrogenesis of the mouse embryonal carcinoma-derived cell line ATDC5. Int J Biochem Cell Biol. 2001;33(11):1094–1103. 11551825 10.1016/s1357-2725(01)00067-x

[45] Scheid MP, Woodgett JR. PKB/AKT: functional insights from genetic models. Nat Rev Mol Cell Biol. 2001;2(10):760–768. 11584303 10.1038/35096067

[46] Peng XD, Xu PZ, Chen ML, et al. Dwarfism, impaired skin development, skeletal muscle atrophy, delayed bone development, and impeded adipogenesis in mice lacking Akt1 and Akt2. Genes Dev. 2003;17(11):1352–1365. 12782654 10.1101/gad.1089403 PMC196068

[47] Fujita T, Azuma Y, Fukuyama R, et al. Runx2 induces osteoblast and chondrocyte differentiation and enhances their migration by coupling with PI3K-Akt signaling. J Cell Biol. 2004;166(1):85–95. 15226309 10.1083/jcb.200401138 PMC2172136

[48] Xiong Y, Zhao B, Zhang W, et al. Curcumin promotes osteogenic differentiation of periodontal ligament stem cells through the PI3K/AKT/Nrf2 signaling pathway. Iran J Basic Med Sci. 2020;23(7):954–960. 32774819 10.22038/IJBMS.2020.44070.10351 PMC7395181

[49] Ye C, Zhang W, Hang K, et al. Extracellular IL-37 promotes osteogenic differentiation of human bone marrow mesenchymal stem cells via activation of the PI3K/AKT signaling pathway. Cell Death Dis. 2019;10(10):753. 31582734 10.1038/s41419-019-1904-7 PMC6776644

