# Supplementary figures and images for: The potential mechanism of Fructus Ligustri Lucidi promoting osteogenetic differentiation of bone marrow mesenchymal stem cells based on network pharmacology, molecular docking and experimental identification
Source: Bioengineered. 2022 Apr 27;13(4):10640–53. doi: 10.1080/21655979.2022.2065753 (PMC9208528; doi:10.1080/21655979.2022.2065753)

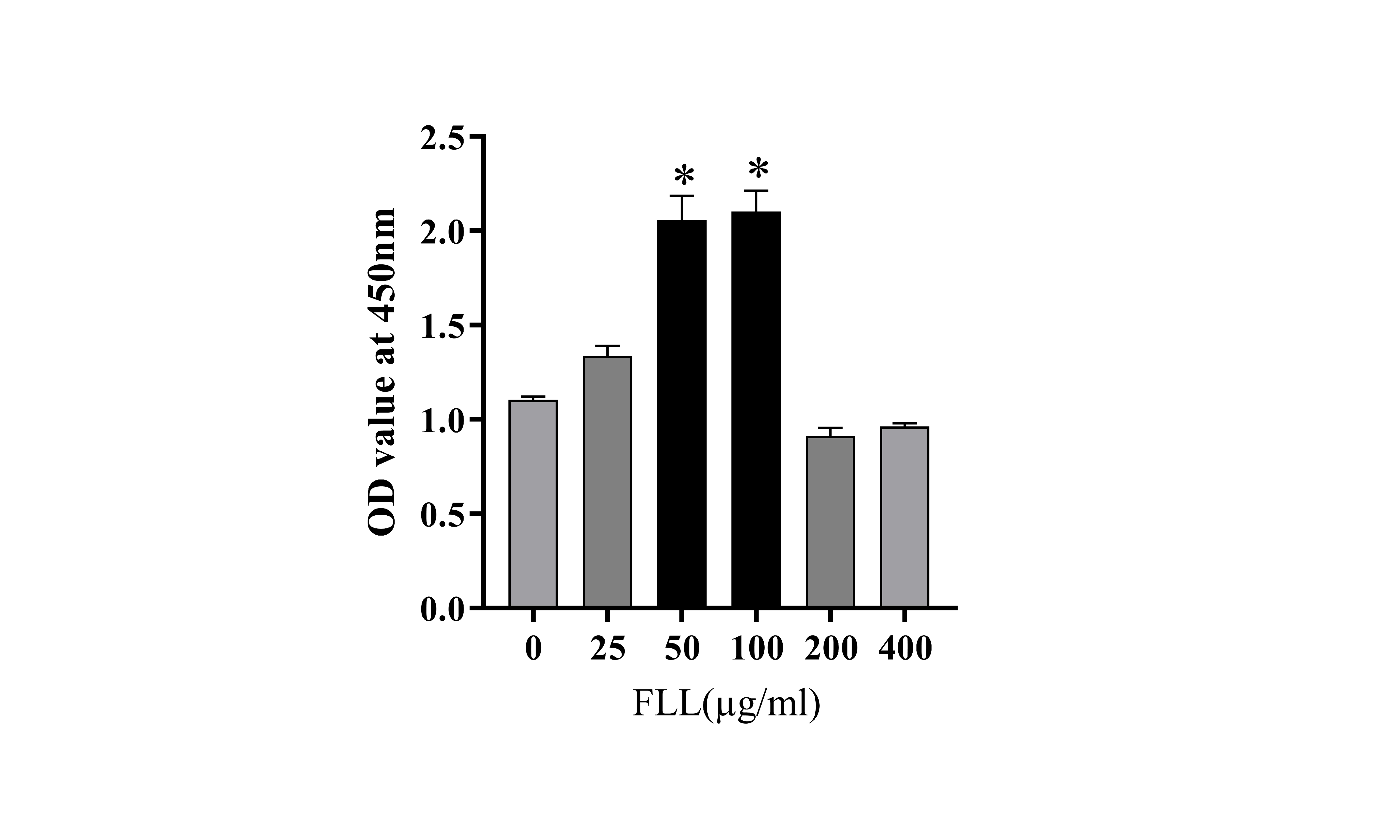

Supplement: Supplemental Material [file KBIE_A_2065753_SM1630.zip › supplementary/FigS1.tif]

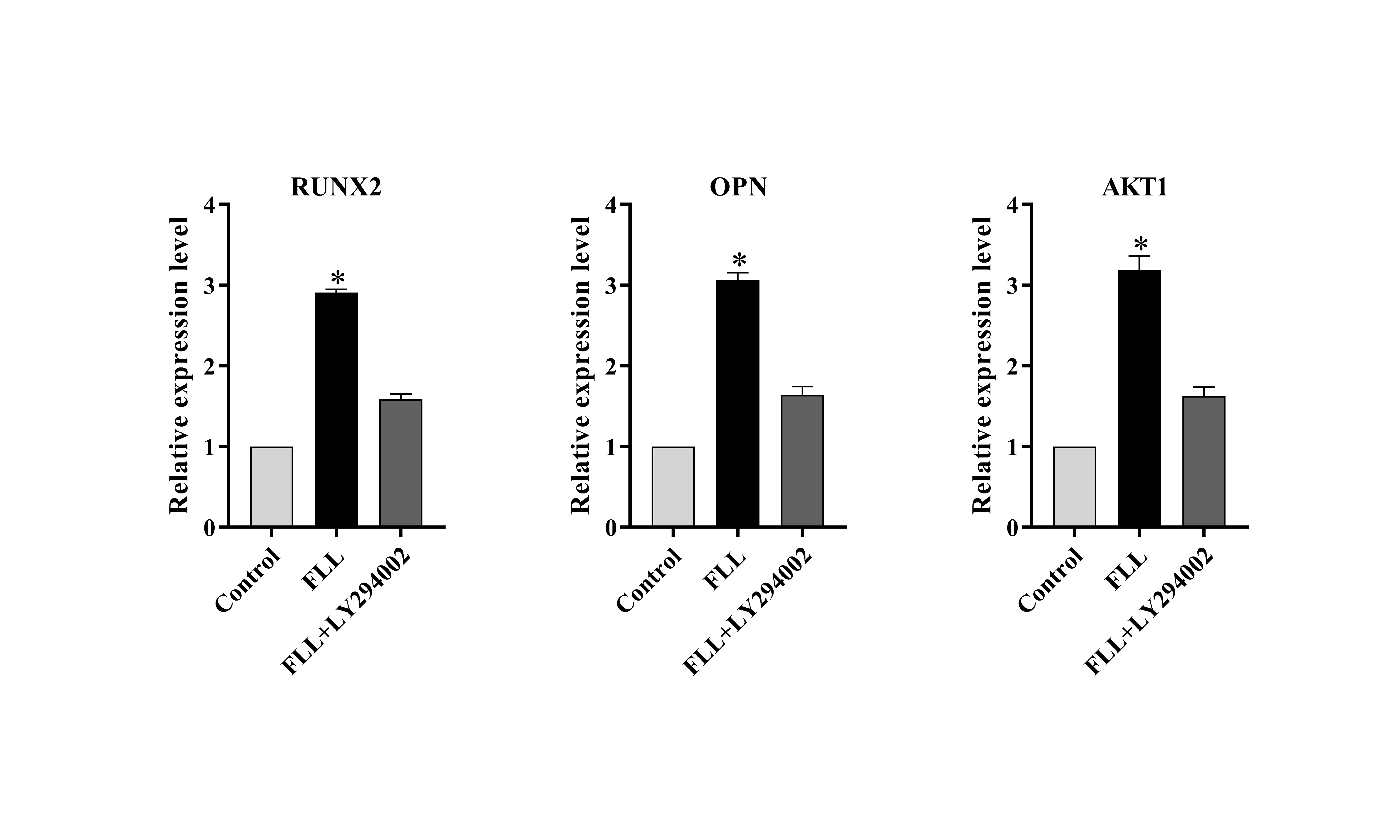

Supplement: Supplemental Material [file KBIE_A_2065753_SM1630.zip › supplementary/FigS2.tif]
